# A Possible Role of the Aleurone Expressed Gene *HvMAN1* in the Hydrolysis of the Cell Wall Mannans of the Starchy Endosperm in Germinating *Hordeum vulgare* L. Seeds

**DOI:** 10.3389/fpls.2019.01706

**Published:** 2020-01-20

**Authors:** Raquel Iglesias-Fernández, Elena Pastor-Mora, Jesús Vicente-Carbajosa, Pilar Carbonero

**Affiliations:** ^1^ Centro de Biotecnología y Genómica de Plantas-Severo Ochoa (CBGP, UPM-INIA), Universidad Politécnica de Madrid (UPM) - Instituto Nacional de Investigación y Tecnología Agraria y Alimentaria (INIA), Pozuelo de Alarcón, Spain; ^2^ Departamento de Biotecnología-Biología Vegetal, Escuela Técnica Superior de Ingeniería Agronómica, Alimentaria y de Biosistemas, Madrid, Spain

**Keywords:** aleurone layer, barley, endo-β-mannanase (MAN), endosperm cell walls, heteromannans, reserve mobilization, grain germination

## Abstract

The barley endo-β-mannanase (*MAN*) gene family (*HvMAN1-6*) has been identified and the expression of its members analyzed throughout different plant organs, and upon grain development and germination. The *HvMAN1* gene has been found to be highly expressed in developing and germinating grains. The MAN (EC 3.2.1.78) enzymatic activity gets a maximum in grains at 48 h of germination (post-germination event). Immunolocalization of mannan polymers in grains has revealed the presence of these polysaccharides in the endosperm cell walls (CWs). By mRNA *in situ* hybridization assays, the *HvMAN1* transcripts have been localized to the aleurone layer, but not to the dead starchy endosperm cells. These data suggest that MAN1 is synthesized in the aleurone layer during early grain imbibition and moves potentially through the apoplast to the endosperm where the hydrolysis of the mannan polymers takes place after germination *sensu stricto*. Hence, mannans in the starchy endosperm CWs, besides their structural function, could be used as reserve compounds upon barley post-germination.

## Introduction

The monospermic fruit of the Poaceae (grain) is made up by the seed and the pericarp (fruit tissue proper). These seeds are formed by a small diploid (2n) embryo and a triploid (3n) endosperm, wrapped together by the maternal tissue of the seed coat (testa, 2n; Linkies et al., 2010). The endosperm is a nutritive tissue that is generated by endoreduplication and during maturation accumulates reserve compounds such as polysaccharides (i.e. starch) and proteins (i.e. hordeins in barley). The endosperm undergoes programmed cell death (PCD) upon maturation, with the exception of its external cell layer (aleurone) that remains alive in the mature grain (Domínguez and Cejudo, 2014).

In barley and other Poaceae, germination *sensu stricto* occurs in two sequential steps where the coleorhiza emergence is followed by the root protrusion. Afterwards, post-germinative events take place, involving the mobilization of reserves from the endosperm, facilitating the early seedling growth until the plant becomes fully photosynthetic. It has been proposed that not only starch and proteins but also other structural polysaccharides, such as β-mannans and β-glucans present in the endosperm cell walls (CWs), could be hydrolyzed and remobilized as reserve compounds to the growing embryo (Barrero et al., 2009; Guillon et al., 2012; González-Calle et al., 2015). This remobilization process needs the synthesis and secretion of appropriate hydrolytic enzymes (mannanases, glucanases, etc.). The *de novo* synthesis of these enzymes is initiated in the scutellum epithelium and later in the aleurone layer (AL). Gibberellin (GA) synthesized in the scutellum is transported to the AL where it activates the synthesis of hydrolytic enzymes that are subsequently secreted into the endosperm (Fincher, 1989; Appelford and Lenton, 1997). The barley α-amylase was the first enzyme described as to be released from the AL to the starchy endosperm, triggered by the embryo secreted GA during seed post-germination (Chrispeels and Varner, 1967). More recently, a late maturity α-amylase has been described in wheat although its transcriptional or hormonal control is not fully elucidated (Barrero et al., 2013). Many other hydrolytic enzymes induced by GA and repressed by abscisic acid (ABA) have been described as involved in reserve mobilization upon monocotyledonous, and dicotyledonous seed germination and post-germination, such as cathepsin β-like cysteine proteases, that are endopeptidases mainly associated to the hydrolysis of seed storage proteins (SSPs; Mena et al., 2002; Isabel-Lamoneda et al., 2003; Martínez et al., 2003; Moreno-Risueno et al., 2007; Tan-Wilson and Wilson, 2012; Iglesias-Fernández et al., 2014; Díaz-Mendoza et al., 2019).

Endo-β-mannanases (MANs; E.C. 3.2.1.78) are enzymes that catalyze the hydrolysis of the β-(1–4) bonds in the mannan polymers. In dicotyledonous seeds, such as those from *Arabidopsis thaliana*, *Brassica rapa*, and *Sisymbrium officinale*. MAN enzymes have been involved in the weakening of the seed-covering layers to facilitate radicle emergence during germination *sensu stricto* (Iglesias-Fernández et al., 2011a; Iglesias-Fernández et al., 2011b; Rodríguez-Gacio et al., 2012; Bewley et al., 2013; Zhang et al., 2013; Nonogaki, 2014; Steinbrecher and Leubner-Metzger, 2017; Carrillo-Barral et al., 2018). In *Arabidopsis*, the importance of MAN enzymes in the germination *sensu stricto* has been further supported by genetic experiments with T-DNA insertion mutants in genes encoding MANs (*AtMAN5*, *AtMAN6*, and *AtMAN7*; Iglesias-Fernández et al., 2011a, 2011b). Interestingly, the presence of mannan polymers has been shown in the mucilage layer of imbibed *A. thaliana*, *B. rapa* and *S. officinale* seeds by fluorescence immunolocalization assays (Lee et al., 2012; Carrillo-Barral et al., 2018). In the monocotyledonous *Brachypodium distachyon*, a model species for temperate cereals, *BdMAN2*, *BdMAN4*, and *BdMAN6* genes have been demonstrated to be relevant during grain germination, and mannans have been immunolocalized to the coleorhiza, disappearing as germination progresses (González-Calle et al., 2015). In *Oryza sativa*, three *MAN* genes (*OsMAN1*, *OsMAN2* and *OsMAN6*) are expressed in germinating grains prior to radicle emergence (Yuan et al., 2007; Ren et al., 2008). In *Hordeum vulgare*, the HvMAN1 protein has been purified from seedling extracts, and the highest expression level of its corresponding transcript is found in the early developing grain (Hrmova et al., 2006). In these grains, mannan polymers have been immunodetected in the CWs of the starchy endosperm, but not in the aleurone cells (Fincher, 2009; Wilson et al., 2012).

In this work, the barley *MAN* gene family has been annotated and the expression of its members analyzed throughout different plant organs, and upon grain development, germination and post-germination. Interestingly, it has been found that the *HvMAN1* gene is highly expressed during grain development and germination, both in the embryo and in the de-embryonated grain (endosperm). MAN enzymatic activity peaks in the germinated barley grain (42–72 h). Mannan polymers have been detected by fluorescence immunolocalization in the endosperm CWs of developing grains. However, in germinating grains the *HvMAN1* transcripts have been localized to the aleurone cells, but not to the starchy endosperm. All together, our data suggest that mannans, deposited in the endosperm CWs during grain development, besides their structural function, could be used as reserve compounds to be mobilized by MAN enzymes upon barley post-germination. Moreover, expression analysis and *mRNA in situ* hybridization assays indicate that the *HvMAN1* is synthesized in the AL during early grain imbibition, bringing up the idea that the MAN1 protein (without excluding other MANs) could move through the apoplast from the aleurone to the endosperm where the hydrolysis of the mannan polymers takes place after germination *sensu stricto*.

## Materials and Methods

### Plant Material and Growth Conditions

Barley (*Hordeum vulgare* cv. Bomi) grains were surface-sterilized (1% NaOCl for 10 min) and germinated in Petri dishes with water imbibed filter papers (Whatman n°3, GE Healthcare Life Sciences, Chicago, IL, USA), at 21°C in the dark for 3 days. After this period, seedlings were transferred to pots in the greenhouse under long-day conditions (16h/8h; light/darkness).

For developmental studies, grains were collected at different stages and classified according to their size and color: White 1 (W1, 2–3 mm), White 2 (W2; 3–4 mm), White 3 (W3; 4–6), Early Green (EG; 6–8 mm), and Late Green (LG; > 8 mm).

For germination experiments, grains after cold-stratification (4°C, 4 days) were imbibed in water in dark conditions at 21°C, sampled at different germination times (0, 4, 8, 12, 24, 48, and 72 h) in triplicate lots of 15–20 stratified grains and separated with a scalpel blade under a magnifying lens into endosperm proper and embryo enriched-fraction (de-embryonated grains; more than 80% of embryo tissue).

### Barley Mannanase (*HvMAN*) Sequence Identification and Phylogenetic Dendrogram Construction

The HvMAN1, HvMAN2, HvMAN3, HvMAN4; HvMAN5, HvMAN6 amino acid sequences have been deduced from their corresponding *MAN* genes in the *Hordeum vulgare* genome (The International Barley Genome Sequencing Consortium, Schulte et al., 2009; accession numbers in **Supplementary Table S1**). For this, the *Arabidopsis thaliana* and *Brachypodium distachyon* MAN sequences (Iglesias-Fernández et al., 2011a; González-Calle et al., 2015) were used as queries in the BLAST tool at the *Ensembl Plant* Server (http://plants.ensembl.org/index.html). The domain glycosyl-hydrolase 5, typically present in MAN enzymes, has been found in all HvMAN amino-acid sequences using the Pfam search tool (PFAM database; http://pfam.sanger.ac.uk; Bateman et al., 2002). Complete amino acid MAN sequences from *Hordeum vulgare* (HvMAN1-6), *Brachypodium distachyon* (BdMAN1-6), and *Arabidopsis thaliana* (AtMAN1-7) were aligned by means of the CLUSTAL W program (Thompson et al., 1994). This multiple alignment was utilized to construct a phylogenetic dendrogram with the MEGA 4.0 software (Tamura et al., 2007) using the neighbor-joining algorithm, a bootstrap analysis with 1,000 replicates, complete deletion and the Jones Taylor Thornton matrix. The identification of conserved motives within the deduced MAN proteins (**Table 1**) was done using the MEME program software version 4.0 and used to validate the phylogenetic tree. Motif width was set between 8–30 amino acids and the maximum number of motives to find was set to 20, the rest of parameters was set as default (Bailey et al., 2009; http://meme-suite.org/). **Table 1** displays the consensus sequences where the amino acids appearing in a given position show a probability >0.2. Signal peptide cleavage sites, isoelectric points (Ip), and molecular weights (MW) were predicted as described (Iglesias-Fernández et al., 2011a; **Supplementary Table S1**).

**Table 1:** Conserved amino acid motives of the MAN deduced proteins of *Hordeum vulgare*, *Brachypodium distachyon* and *Arabidopsis thaliana*, obtained by means of the MEME analysis (Bailey et al., 2009).

Motif	E-value	Consensus sequences
**1**	6.8e-390	[VL]L[TN]R[VK]N[TS][FILV]TG[VI]AYKD[DE]P[TA]I[FL]**AWEL[MI]NEPRC**
**2**	3.1e-225	[IL]L[SC]LVNN[LWY][DE][DA][FY]GGK[KAT]QYV[KQR]WA
**3**	5.5e-214	LQ[AD]W[IV][ET]EMA[AS][YF]VKS[IL]DX[KN]H[LM][LV]
**4**	1.2e-285	[AP]LQI[SA]PG[VRS][YF][DN]E[ER][VM]F[QK][GA]LDFV[IVL][AS]EA[RK][RK]HG[IV][RKY]
**5**	3.1e-273	[VLY]GTDF[IV][AR]N[NHS]Q[VIA]PGIDFA[ST][VI]H[SIL]YP**D**[QS]W[LF]P[DG][SA]
**6**	4.9e-226	RG[KR]V[TS][AE][AM][LF][RQ][QA][AG][AS][AR][MH]GL[TN]V[CA]RT**W**AF[NS]DGG[YS][NR]
**7**	4.9e-192	G[TV][QR]FV[LV][NG]G[RK]PFY[AI]NG[FW]N[AS]YWLM[YDT]MA[ASV][DE]P[AS]
**8**	1.1e-165	S[DN]D[DS]FF[TF][DN]P[LT][IV][KR][DG][YF][YF]KN[HY]VK[TA]
**9**	1.3e-103	[ET][IVA]GLEGFYG[PD][SG][SA]PE[RS]
**10**	1.6e-098	E[ES][QK][LV][EK]F[VL]R[KR]W[ML][DAQ][SA]H[IV]ED[AG][EAQ]N[IE]
**11**	2.0e-072	[KRG]KP[LV]L[FILV][TA]**E**FG[KL]
**12**	1.5e-070	L[FV]**W**Q[LV][MF]AEGMEX[FY]HD
**13**	8.9e-113	RDAF[FLY][RG][TM]VYD[KA]IY[AE]SA[KRE][KR]GG[PAS]
**14**	1.7e-075	G[FY]SIV[LAP]SEX[PA]STAK[LI]I[ST]E[QH]S[CR][RK]LAXLR[KG]
**15**	9.6e-012	[CRT][RN][EGN][LR][FPR][LV]Y[HS]ILG[FL][LA][LS][LC][LV]A[VF][FIV]Y[LF][SN]
**16**	2.2e-007	PVPWLQPRM[AS]FAGRNGTHFVDA[AS]TG[AS]PLYV
**17**	5.8e-007	L[GT][VF]NP[GDE][DH][WY][AFS][AGNS][NQS]
**18**	2.7e-004	MK[CL]L[CA][FL][FIV][VP][LF]LAI[VL]I[AQ][QL][SN][CSY]

MAN signature sequence is underlined and in bold. Critical amino acids for enzymatic activity are displayed in bold.

### Endo-β (1-4)-Mannanase (MAN) Activity Assays During Grain Germination

*Hordeum vulgare* grains were collected in triplicate lots of 20 individuals at different time-points of germination (0, 12, 24, 48, 72 h). Grains were separated into embryo and endosperm fractions and then frozen in liquid nitrogen. Samples were homogenized in 100 mM NaAc buffer (pH 4.7) overnight at 4°C and subsequently centrifugated. The supernatant (400 μl) was mixed with 200 μl of 0.25% AZCL galactomannan (Megazyme, Chicago, IL, USA). Enzymatic activity was determined as previously described (Iglesias-Fernández et al., 2011a; Carrillo-Barral et al., 2018). For quantification of total protein, the Bradford reagent was utilized (Sigma-Aldrich, Merck KGaA, Darmstadt, Germany) and Bovine Serum albumin (BSA) was the standard.

### Total RNA Isolation and Quantitative PCR Analyses

Total RNA was purified from leaves, roots, spikes, and stems of 7 week old plants and from grains at different stages of development (W1, W2, W3, EG, and LG) and different points of germination (0, 4, 8, 12, 24, and 48 h) using the protocol described by Oñate-Sánchez and Vicente-Carbajosa (2008). RNA samples were treated with DNAseI-RNAse-free (Hoffmann-La Roche, Basel, Switzerland) to avoid genomic DNA contamination. RNA integrity and purity were checked both electrophoretically and by the 260/280 nm absorbance ratio. The RNA concentration was estimated by the *A*_260_ absorbance, and the samples were stored at –80 °C. The cDNA was synthesized from 1 μg of total RNA using the First-Strand Synthesis kit for RT-PCR (Hoffmann-La Roche) and stored at – 20°C until used.

For gene expression analysis, PCR was performed in an Eco Real-Time PCR System (Illumina, San Diego, CA, USA). In each reaction (V_f_ = 10 µl) were mixed: 2 µl of cDNA sample, 5 µl of FastStart SYBR Green Master (Hoffmann-La Roche), 0.25 ul of each primer (final concentration 500 nM), and sterile water up to final volume. The PCR thermal-cycling conditions were set as follows: 95°C for 10 min for denaturation and 40 cycles of 10 s at 95°C and 30 s at 60°C for annealing and extension. The dissociation temperature for each amplicon was calculated by increasing temperature from 55 to 95°C. The dilution curve and subsequent slope calculation (E = 10^(-1/slope)^) were used to calculate primer efficiencies (**Supplementary Table S2**). The specific primers used were designed from the specific 3’-non-coding region using the Primer3Plus program (http://www.bioinformatics.nl/cgi-bin/primer3plus/primer3plus.cgi; **Supplementary Table S2**). The *HvGAPDH* gene (encoding GlycerAldehyde 3-Phospate DeHydrogenase; HORVU7Hr1G074690) was used to normalize the data, since the expression of this gene is constant throughout the period studied (**Supplementary Figure S1**; González-Calle et al., 2015; Gines et al., 2018). The number of cycles required for the amplification to get a cycle threshold in the exponential phase of the PCR (C_t_; Pfaffl, 2001) was used to calculate the expression levels. All analyses were done in three biological samples and two technical replicates.

Statistical analysis of the data was performed in Excel (version 2007) using the t-test for comparing the experimental data with the minimum levels of each experiment.

### Fixation, Embedding and Sectioning of Material for Histochemistry

For the histochemical analysis, LG stage of developing barley grains and germinating grains at 4, 30, and 42 h of imbibition were processed, as described by Ferrándiz et al. (2000) with some modifications. Grains were collected and infiltrated with the FAE solution (Formaldehyde: Acetic acid: Ethanol: water, 3,5:5:50:41,5 by vol.) for 45 min under vacuum (41 mbar) then incubated at 4°C for 3 days with gentle shaking. A graded series of aqueous ethanol mixtures were used to dehydrate the grain samples, ethanol being progressively replaced with HistoClear (National Diagnostics, Hessle Hull, England) and later embedded in paraffin. Thin sections of 8–10 µm were collected on glass slides and de-waxed.

### Fluorescent Heteromannan Immunolocalization

Heteromannan immunolocalization was performed as described by Marcus et al. (2010) and Guillon et al. (2012). Sections were first washed with Phosphate Buffer Sodium solution (PBS) and digested with 1 mg/ml proteinase-K (Hoffmann-La Roche). Samples were then treated with 4 µg/ml lichenase [(1-3) (1-4)-β-glucanase; Megazyme] for 2 h at 37°C to eliminate (1–3) (1–4)-β-glucans and thus facilitate the access of the specific antibodies to the heteromannans (mannans, glucomannans, and galactomannans) contained in the CWs. For immunodetection, sections were first incubated at room temperature for 30 min in a blocking solution (3% BSA, 1x PBS, and 5mM Na-Azide; pH7), and then treated with primary anti-heteromannan antibody LM21 (Plant Probes, Leeds, UK) at a dilution of 1:5 in 1% BSA for 2 h. Sections were thoroughly washed in PBS containing 5 mM Na-Azide and then incubated for 2 h in the same buffer containing the secondary rabbit antibody Anti-Rat IgG-FITC (Sigma-Aldrich) at a dilution of 1:100. The sections were extensively washed in PBS buffer and in water, mounted in citifluor (Citifluor, Hatfield, PA, USA) and examined in a confocal microscope (Leica SP8, Leica, Wetzlar, Germany).

### *mRNA In Situ* Hybridization Experiments

Plant sections were incubated in 0.2 M HCl and, after washing, digested with 1 mg/ml proteinase-K (Hoffmann-La Roche). Samples were then dehydrated in an aqueous ethanol dilution series and hybridized with sense and anti-sense digoxigenin (DIG)-labelled RNA probes, corresponding to DNA fragments (200–300 bp) derived from the specific 3’-non coding regions of the *HvMAN1* gene (**Supplementary Table S3**), synthesized according to the manufacturer’s instructions (Hoffmann-La Roche). Probes were hybridized at 52°C overnight followed by two washes in 2X SSC (150 mM NaCl, 15 mM Na_3_ Citrate) and 50% formamide for 90 min at the same temperature. Incubation with the alkaline phosphatase-conjugated anti-digoxigenin antibody (Hoffmann-La Roche) and color detection was carried out according to the manufacturer’s instructions. Sections were dried and examined on a Zeiss Axiophot Microscope (Carl Zeiss, Oberkochen, Germany), images were captured and processed with the Leica Application Suite 2.8.1 build software (Leica, Wetzlar, Germany).

### Polysaccharide and Protein Histological Staining for Light Microscopy

Developing and germinating grains were stained with PAS-NBB: 0.5% (w/v) Periodic Acid (Merck) plus Schiff’s reagent (Merck) to detect polysaccharides, and with 1% (w/v) Naphthol Blue Black (Sigma-Aldrich) to visualized proteins. Microscopy analyses were done on a Zeiss LSM 880 (Carl Zeiss) microscope and the images were captured and processed with the Zen Blue Edition software (Carl Zeiss).

## Results

### The *Hordeum vulgare* Endo-β-Mannanase (*HvMAN*) Gene Family

**Figure 1:**
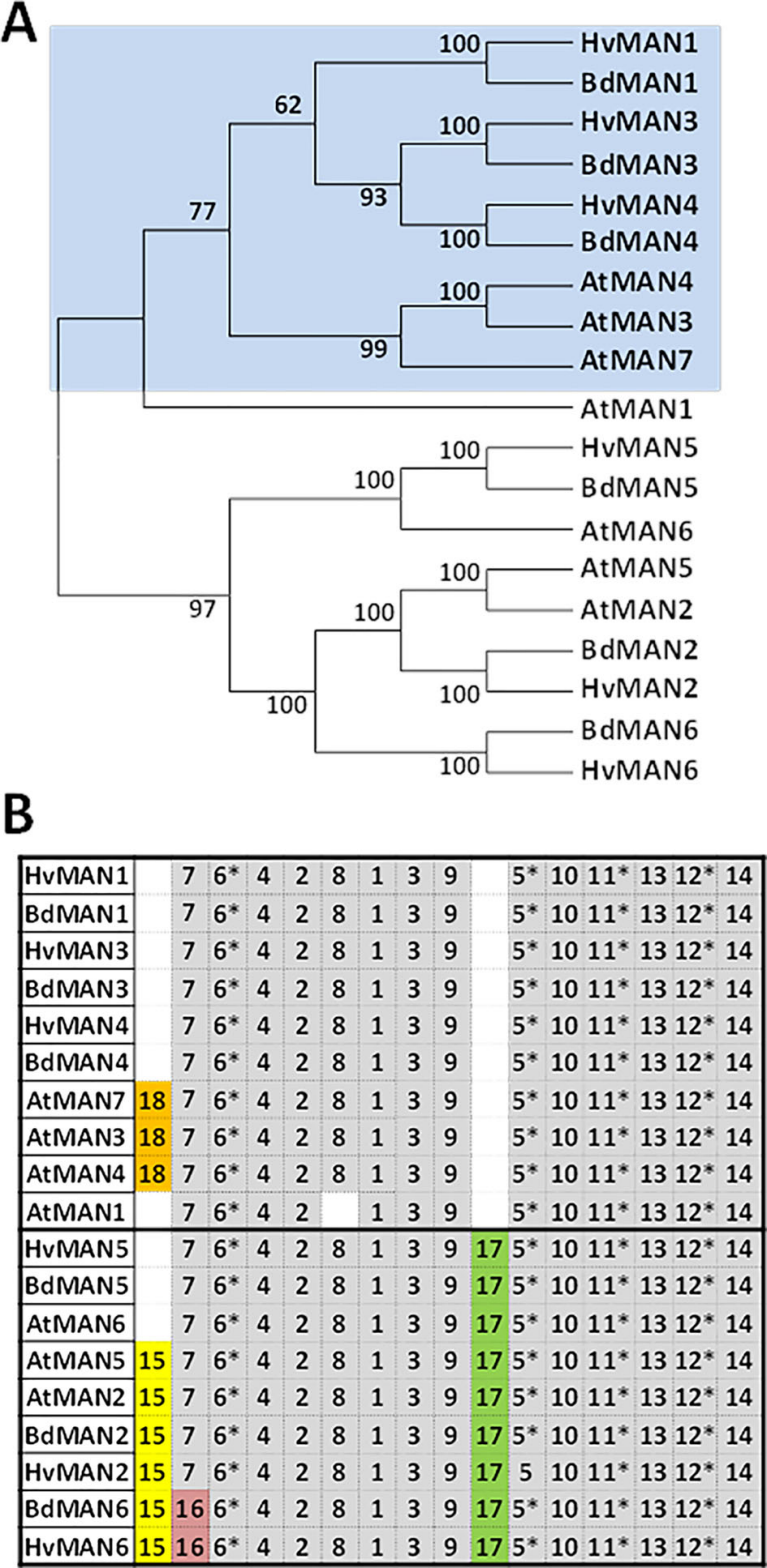
The *MAN* gene family in *Hordeum vulgare* **(A)** Phylogenetic dendrogram of the deduced amino-acid sequences of *Hordeum vulgare*, *Brachypodium distachyon*, and *Arabidodpsis thaliana MAN* gene families; numbers in branches indicate bootstrapping values. **(B)** Schematic distribution of conserved motives among the deduced protein sequences in the phylogenetic dendrogram in **(A)**, identified by the MEME analysis. Asterisks show motives that include amino-acids involved in the catalytic centre of the enzyme. Motives in grey are conserved in more than 85% of the sequences.

To identify the different *HvMAN* genes in barley, sequences of the already described *MAN* families from *Arabidopsis thaliana* and *Brachypodium distachyon* (Iglesias-Fernández et al., 2011a; González-Calle et al., 2015) were used to do a BLASTN against the whole *Hordeum vulgare* genome (http://plants.ensembl.org/index.html; The International Barley Genome Sequencing Consortium). Six non-redundant MAN deduced proteins were identified and named according to their putative orthologous in *B. distachyon*, with MW between 46–54 KDa and Isoelectric points between 4.6–9.2. Four of them (HvMAN1, HvMAN3, HvMAN4, and HvMAN6) have N-terminal predicted signal peptides (**Supplementary Table S1**). The MAN protein sequences from Brachypodium (BdMAN1-6), Arabidopsis (AtMAN1-7) together with those from *Hordeum vulgare* were used to construct an unrooted dendrogram by using the neighbor-joining algorithm. Two main clusters of orthologs could be found, supported by bootstrapping values higher than 62% (**Figure 1A**) and by the occurrence of common motives (**Figure 1B**).

The search of conserved amino-acid motives using the MEME software (http://meme-suite.org/) shows that all the MAN sequences have in common several motives described as critical for the enzymatic activity, such as 5, 6, 11, and 12 (**Figure 1B** and **Table 1**). The deduced signature sequence [AWEL(MI)NERPRC] described for Arabidopsis MAN proteins (Yuan et al., 2007), included in motif 1, is also present in the *Hordeum vulgare* MAN proteins annotated (**Table 1**). Besides, MAN2, MAN5, and MAN6 share motif 17, that is lacking in MAN1, MAN3, MAN4, and AtMAN7. Motif 15 is found in AtMAN2, BdMAN2, HvMAN2, BdMAN6, HvMAN6, and AtMAN5, but not in HvMAN5, BdMAN5, and AtMAN6, although these last three MAN proteins are included in the same tree branch, supported by a bootstrap value of 100. Motif 16 is present in BdMAN6 and HvMAN6 supporting a branch with a bootstrap value of 100 (**Figure 1B**).

### *HvMAN1* Is Highly Expressed Upon Grain Development and Endosperm Cell Walls (CWs) Are Rich in Mannan Polymers

**Figure 2:**
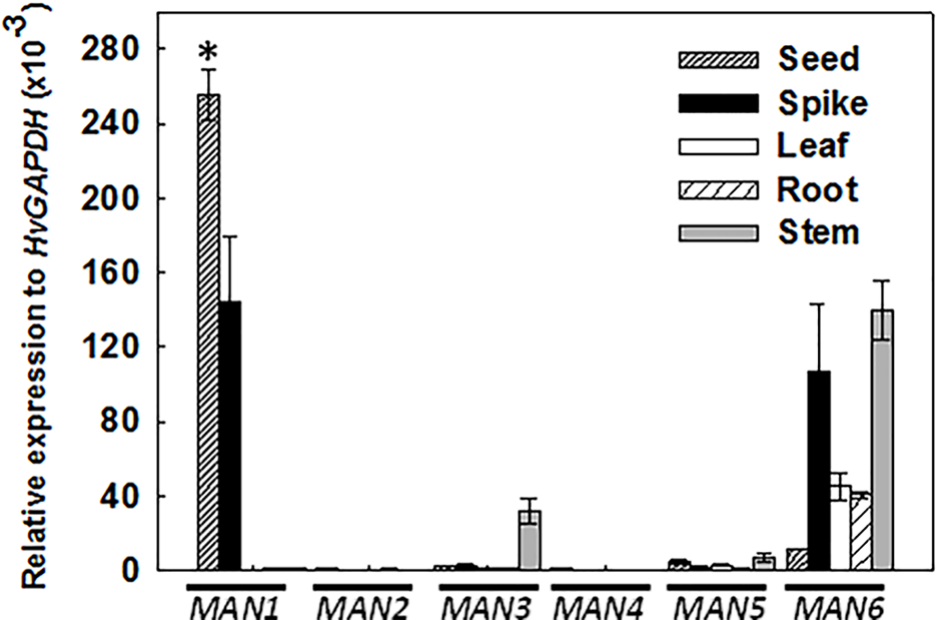
Expression analysis of the *HvMAN* members in several organs of barley plants. Transcript accumulation of *HvMAN1*, *HvMAN2*, *HvMAN3*, *HvMAN4*, *HvMAN5*, and *HvMAN6* in reproductive (grains and spikes) and vegetative (leaf, root, and stem) organs of 7-week-old plants, evaluated by quantitative PCR. Data are means ± standard error (SE) of three biological and two technical replicates. Asterisks indicate significant differences (P < 0.05) as determined by the t-test. Gene expression in different organs has been compared with the corresponding data of the *HvMAN6* gene.

The expression pattern of the *HvMAN1-6* genes has been explored by quantitative PCR in vegetative (leaf, root, and stem) and in reproductive organs (grain, spike). As shown in **Figure 2**, *HvMAN1* and *HvMAN6* are the only members of the *HvMAN* family with significant expression levels in the samples analyzed. *HvMAN1* is the most expressed *MAN* gene in grain and spike, reaching ~260 and ~140 relative expression to *HvGAPDH* (x10^-3^), respectively. *HvMAN1 transcripts* are not observed in leaves, root, and stems. *HvMAN6* transcripts are abundant in spikes, leaves, roots, and stems. However, *HvMAN2*, *HvMAN3*, *HvMAN4*, and *HvMAN5* are faintly expressed in the organs analyzed, with the exception of *HvMAN3*, whose transcripts are detected in stems (~30 relative expression to *HvGAPDH* x10^-3^).

**Figure 3:**
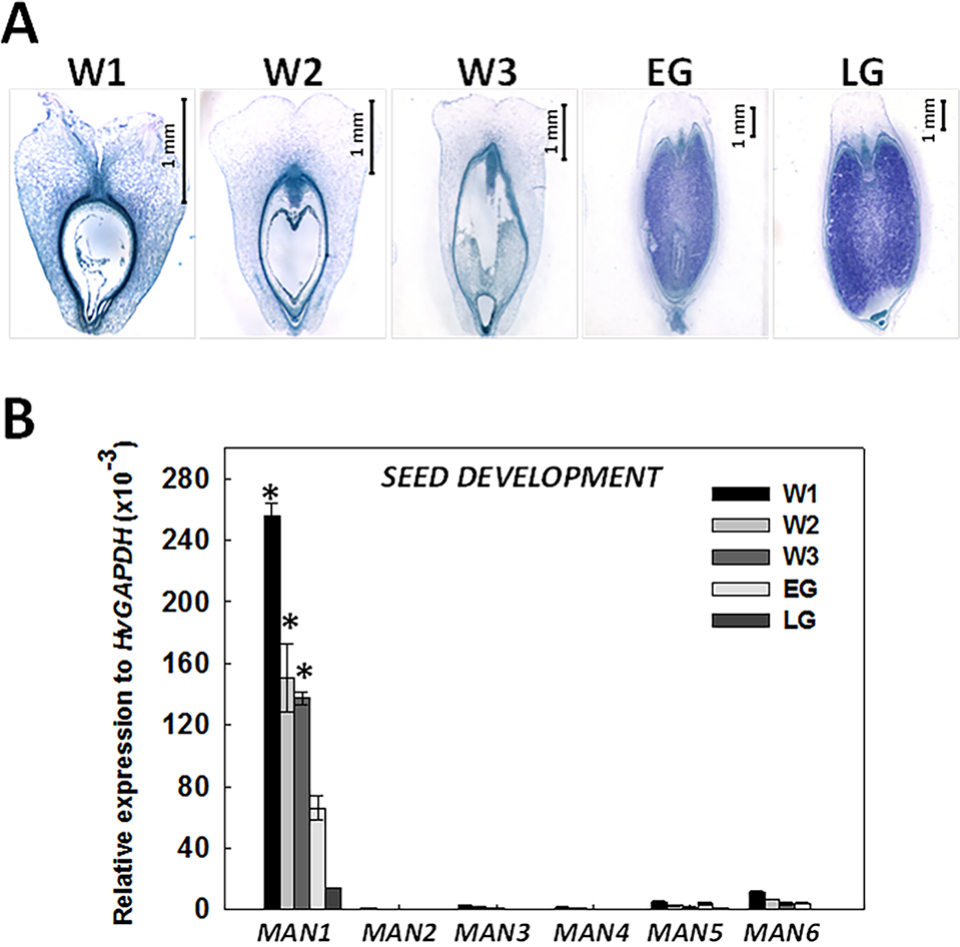
Expression analysis of the *HvMAN* gene family upon barley grain development. **(A)** Stages of grain development in barley stained with NBB-PAS: White 1 (W1: 1 day after pollination, DAP), White 2 (W2: 5 DAP), White 3 (W3: 10 DAP), Early Green (EG: 16 DAP), and Late Green (LG22 DAP; Druka et al., 2006) **(B)** Transcript accumulation of *HvMAN1*, *HvMAN2*, *HvMAN3*, *HvMAN4*, *HvMAN5*, and *HvMAN6* upon the different grain developmental stages described in **(A)**. Data are means ± standard error (SE) of three biological and two technical replicates. Asterisks indicate significant differences (P < 0.05) as determined by the t-test. Expession levels in different developmental stages were compared with the corresponding data of the LG stage.

Considering the results of **Figure 2**, we further analyzed the expression profile of the *HvMAN1-6* during different stages of grain development, previously defined (see *Materials and Methods* and **Figure 3A**). In **Figure 3B**, *HvMAN1* is the only member of the *HvMAN* family expressed throughout grain development. The results clearly show a decrease in the level of transcripts from W1 to LG [W1: ~260; LG: ~10 relative expression to *HvGAPDH* (x10^-3^)]. The transcripts of the other *MAN* genes (*HvMAN2-6*) are faintly detected across the grain developmental stages studied.

**Figure 4:**
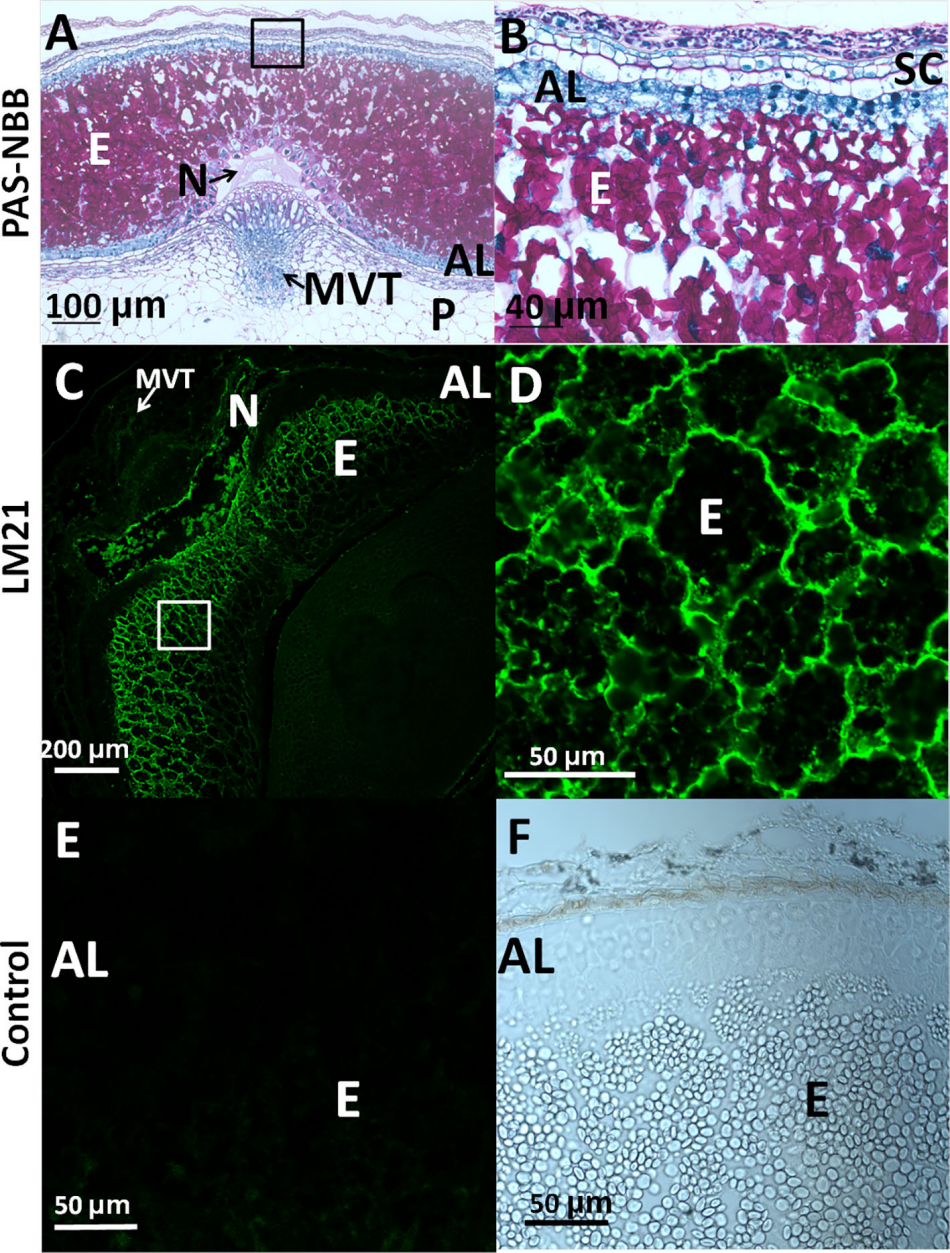
Heteromannan immunolocalization in barley grains at Late Green (LG) stage. **(A)** Polysaccharide and protein detection by bright field microscopy in sections stained with periodic acid (PAS)- Naphthol Blue Black (NBB). Pink: Insoluble polysaccharides; Blue: proteins **(B)** Close-up of the endosperm and aleurone layer cells in **(A)**. **(C)** Mannan polymers immunolocalization in endosperm of barley developing grains using the LM21 specific antibody. **(D)** Close-up of the endosperm cells in 3. **(E)** Secondary Antibody negative control in endosperm and aleurone layer. **(F)** Brighy field image of the aleurone layer and endosperm. AL, Aleurone Layer; E, Endosperm; MVT, Main Vascular Tissue; N, Nucellus; P, Pericarp; SC, Seed Coat.

In order to analyze the presence of mannan polysaccharides in barley grains during development, heteromannan immunolocalization was performed using specific antibodies. With the purpose of evaluating cell and tissue integrity, transversal sections (8 μm) of barley developing grains at LG stage were stained with PAS reagent to detect insoluble polysaccharides (mainly cellulose and starch) and with Naphthol Blue Black (NBB) for proteins. As shown in **Figures 4A, B**, not only cell and tissue integrity has been kept, but it is also shown that starch grains (pink stained) are abundant, and the AL cells are enriched with proteins (blue stained). Mannan polymers have been detected in sections at the LG stage by *in situ* immunofluorescence labelling, using the LM21 antibody (Lee et al., 2012; González-Calle et al., 2015) that specifically recognizes mannan polysaccharides (gluco- and galactomannans). As shown in **Figures 4C, D**, heteromannans are mainly localized to the endosperm CWs, specifically to those cells closer to the outer side of the grain and not to the AL CWs (black). Interestingly, these mannans are barely detected in early stages of grain development (W1; data not shown). Secondary antibody negative controls do not show fluorescence signals as expected (**Figures 4E, F**).

### *HvMAN1* Expression Kinetics and Enzymatic Endo-β-Mannanase Activity During Barley Grain Germination

**Figure 5:**
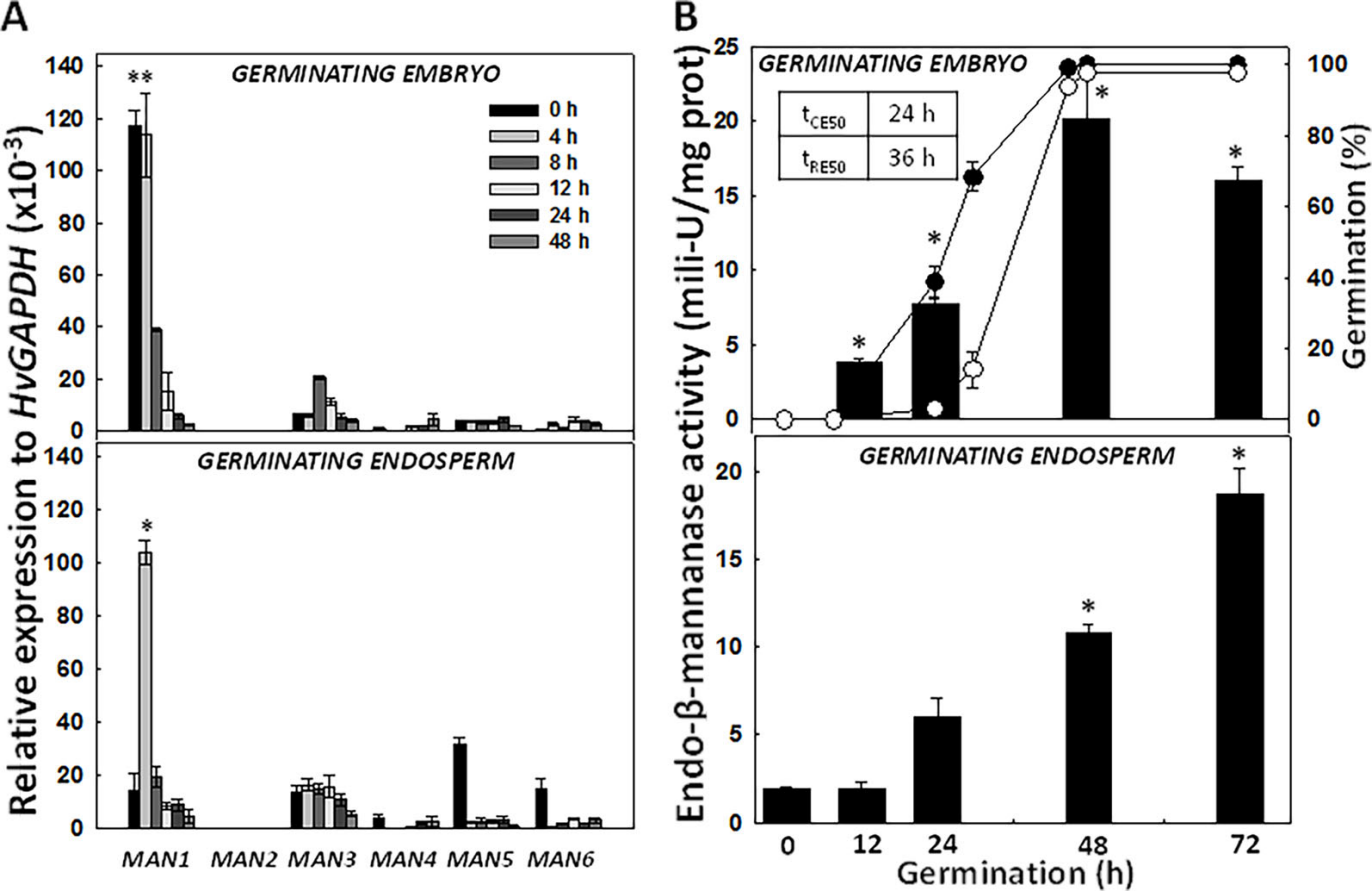
Expression analysis of the *HvMAN* gene family and endo-β-mannanase enzymatic activity at different time-points of barley grain germination. **(A)** Transcript accumulation of *HvMAN1*, *HvMAN2*, *HvMAN3*, *HvMAN4*, *HvMAN5*, and *HvMAN6* in embryos and de-embryonated grains (endosperms) at 0, 4, 8, 12, 24, 48 h of germination. **(B)** Endo-β-mannanase activity (black bars) in embryos and de-embryonated grains (endosperms) at 0, 12, 24, 48, and 72 h of germination. Percentage of germination is represented as coleorhiza emergence (CE; close circles) and root emergence (RE; open circles) Data are means ± standard error (SE) of three biological and two technical replicates. Asterisks indicate significant differences (P < 0.05) as determined by the t-test. Each gene and time-point has been compared with the corresponding data at 0 h.

The expression kinetics of the *HvMAN* genes upon grain germination (0, 4, 8, 12, 24, 48 h) was explored by quantitative PCR (**Figure 5A**). The analysis was done separately in embryos and in de-embryonated grains (endosperms). In dry embryos and at early imbibition stages (4 h), *HvMAN1* is the most highly expressed *MAN* gene [~120 relative expression to *HvGAPDH* (x10^-3^)] and this expression progressively decreases until it disappears at 48 h of imbibition. *HvMAN3* can also be detected upon germination at very low levels. In endosperms, *HvMAN1* is again the most expressed *MAN* gene, peaking at 4 h of imbibition [~120 relative expression to *HvGAPDH* (x10^-3^)], sharply decreasing thereafter. However, *HvMAN3*, *HvMAN5*, and *HvMAN6 mRNAs* are moderately expressed in endosperms during germination. *HvMAN3* is expressed <20 units in all germination stages.

The germination *sensu stricto* in *Hordeum vulgare* occurs in two sequential steps, where coleorhiza emergence (CE; t_CE50_ = ~ 24 h) is followed by root emergence (RE; t_RE50_ = ~ 36 h; **Figure 5B**). After RE, post-germination events take place. MAN activity has been evaluated in embryos and endosperms of germinating grains (0, 12, 24, 48, and 72 h; **Figure 5B**). In embryos, the MAN activity increases as germination progresses, acquiring a maximum at 48 h (~ 20 mili-U/mg prot). In endosperms, MAN activity also progressively increases reaching a maximum at 72 h (~ 20 mili-U/mg prot.), a clear post-germination stage.

### Heteromannans Are Localized to Endosperm Cell Walls (CWs) in Germinating Barley Grains

Mannan polymers could be detected in sections of germinating grains (4, 30, 42 h) by *in situ* immunofluorescence labelling, using the LM21 antibody that specifically recognizes gluco- and galacto-mannans.

**Figure 6:**
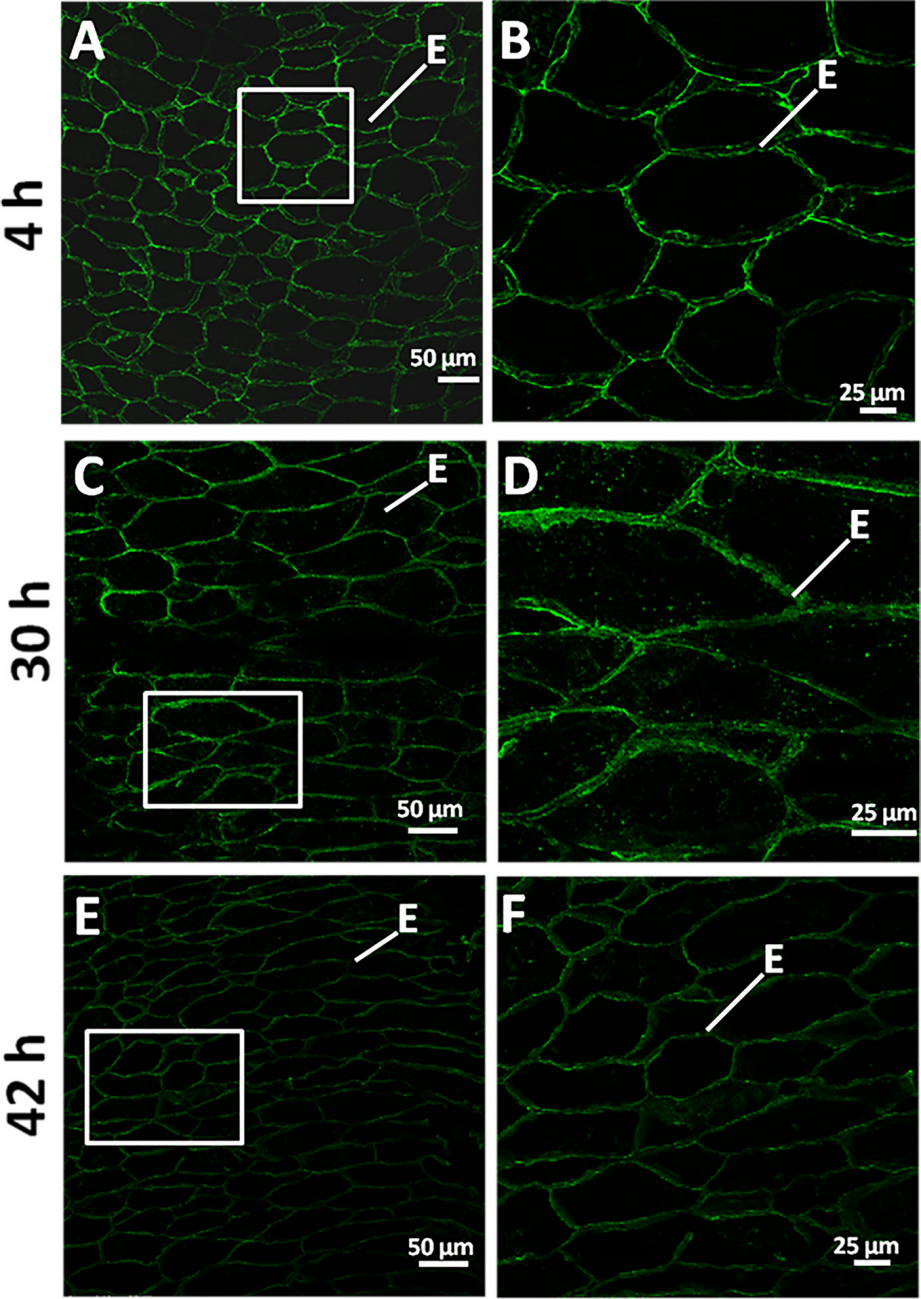
Heteromannan immunolocalization in barley germinating grains. **(A)** Mannan polymers immunolocalization in sections of 4 h germinating grains. **(B)** Close-up of the endosperm cells in **(A)** (white square). **(C)** at 30 h of grain germination. **(D)** Close-up of the endosperm cells in **(C)** (white square). **(E)** at 42 h of grain germination. **(F)** Close-up of the endosperm cells in **(E)** (white square). E, Endosperm.

As it is shown in **Figure 6**, at 4 and 30 h of grain germination, heteromannan polymers are preferentially localized to the endosperm CWs (**Figures 6A–D**). Interestingly, mannans are faintly detected at later stages of germination (42 h; **Figures 6E, F**). Secondary antibody negative controls do not show fluorescence signal as expected (**Supplementary Figures S2A–L**).

### *HvMAN1* Transcripts Are Localized to the Aleurone Cells, to the Embryo and to the Vascular Elements Upon Barley Grain Germination

**Figure 7:**
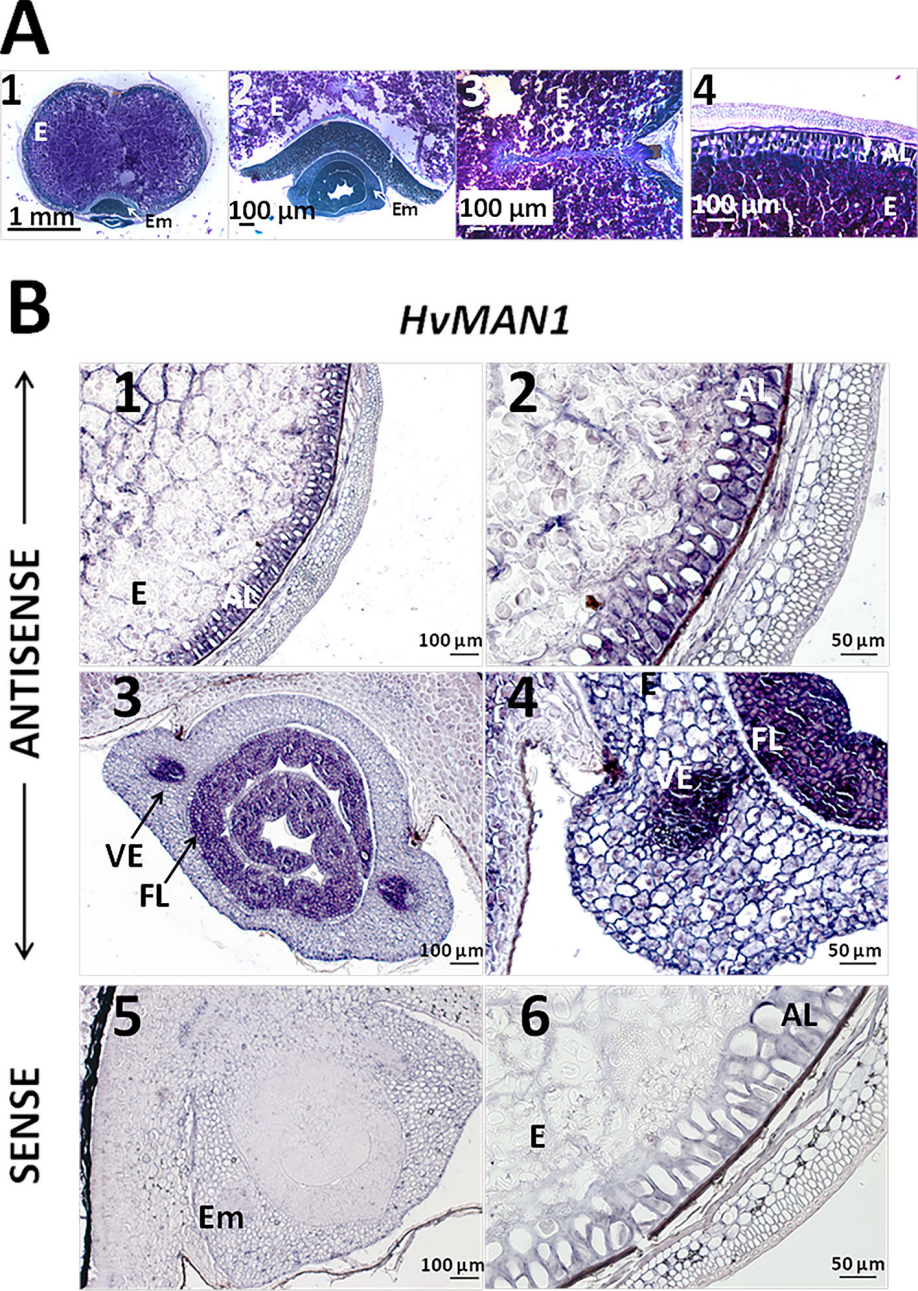
PAS-NBB staining and mRNA *in situ* hybrydization assays of gene *HvMAN1* in germinating barley grains **(A)** Polysaccharide and protein detection in barley germinating grains (30 h) **A1**. Bright field microscopy in transversal sections stained with periodic acid (PAS)- Naphthol Blue Black (NBB). **A2**. Close-up of the embryo. **A3**. Close-up of the endosperm. **4**. Close-up of the aleurone layer. **(B)** *mRNA in situ* hybridization of *HvMAN1* transcripts in germinating grains (30 h). B1. Grain section of the endosperm and the aleurone layer. **B2**. Close-up of the aleurone layer. **B3**. Grain transversal section of the embryo. **B4**. Close-up of the embryo and vascular element in B.3. **B5**. Grain transversal section of the embryo hybridised with the sense probe. **B6**. Detail of the aleurone layer hybridised with the sense probe. AL, aleurone Layer; Em, Embryo; E, Endosperm; FL, First Leaves.

Since our preliminary data showed that *HvMAN1* is the most expressed MAN gene upon grain germination (**Figure 5A**), we focused our analysis in this gene and performed *mRNA in situ* hybridization assays in germinating grains (30 h). In order to evaluate cell and tissue integrity, transversal sections (8 μm) of these barley samples were stained with PAS reagent to detect insoluble polysaccharides (mainly cellulose and starch, pink) and with NBB for protein detection (blue). As shown in **Figures 7A1–4**, cell and tissue integrity was confirmed, and it was also shown that at 30 h of germination starch grains are still abundant in the endosperm, while the AL cells are enriched in proteins (blue stained). *In situ mRNA* hybridization assays, showed that *HvMAN1* transcripts localized to the AL and the vascular elements (VE) in the mesocotyl, and to the first leaves (FL) of the growing embryo (**Figures 7B1–4**). No signal was found in sections treated with the sense probe (**Figures 7B5** and **6**).

## Discussion

In this work, the *MAN* gene family of *Hordeum vulgare* has been annotated and the complete sequence of their six corresponding proteins (HvMAN1-6) used to construct a phylogenetic tree. The expression of the *HvMAN* transcripts has been explored in different vegetative and reproductive organs, mainly in the developing and germinating grains. To determine in which organs (leaves, roots, spikes, stems) of each *HvMAN* family member is expressed, we have used seven-week-old *H. vulgare* plants. *HvMAN1* has been found to be the more abundantly expressed *MAN* gene in reproductive organs (whole grain and spikes), although *HvMAN3*, *HvMAN5*, and *HvMAN6* are also expressed at a lower level in germinating grains. Interestingly, *HvMAN1* is the most expressed gene throughout zygotic embryogenesis, clearly decreasing from phase W1 (high mitotic activity) to LG in which the caryopsis is preparing for desiccation and for the establishment of dormancy (Nonogaki et al., 2018). In other crop and wild species, several *MAN* paralogs have also been described as functionally relevant in grains: *Brachypodium distachyon* (*BdMAN1*, *BdMAN2*, *BdMAN4*, and *BdMAN6*), *Oryza sativa* (*OsMAN1*, *OsMAN2*, and *OsMAN6*), *Arabidopsis thaliana* (*AtMAN2*, *AtMAN5*, *AtMAN6*, and *AtMAN7*), *Brassica rapa* (*BrMAN2*, *BrMAN5*, *BrMAN6*, and *BrMAN7*), *Sisymbrium officinale* (*SoMAN2*, *SoMAN5*, *SoMAN6*, and *SoMAN7*), and *Solanum lycopersicum* (*LeMAN1* and *LeMAN2*; Nonogaki et al., 2000; Nonogaki et al.,2007; Yuan et al., 2007; Ren et al., 2008; Iglesias-Fernández et al., 2011a; Iglesias-Fernández et al., 2011b; González-Calle et al., 2015; Carrillo-Barral et al., 2018).

Although MAN hydrolytic activity is not detected upon embryogenesis and grain maturation (data not shown), heteromannans are immunolocalized to the endosperm CWs at late stages of grain development (LG), but not to the AL (**Figure 4**; Wilson et al., 2012). Endosperm transfer cells may provide activated D-mannose that could be used to synthesize glucomannans and galactomannans present at the barley endosperm CWs (Reiter and Vanzin, 2001; Weschke et al., 2003; Thiel, 2014). Since cell division in the grain phase W1 is important, CW synthesis and remodelling are required (Malinowski and Filipecki, 2002; Gubatz et al., 2007; Rancour et al., 2012). Therefore, the existence of mannan metabolism in these processes cannot be ruled out (Verbančič et al., 2018). On the other hand, MAN enzymes have not only been characterized as hydrolases, but also as mannan endo-transglycolsylases, being this latter activity more related with CW expansion (Schröder et al., 2009). The endo-transglycosylase activity could justify the high expression of *HvMAN1* in W1 phase in absence of heteromannans and mannan hydrolytic activity. It could be possible that *HvMAN1* transcripts expressed at early stages of grain development, and their translated proteins will be involved in cell wall expansion (Sreenivasulu et al., 2010).

In germinating grains, *HvMAN1* is highly induced at early stages and its transcripts are localized to the AL and to the first leaves of embryo (probably related to CW expansion, **Figure 7B**), but not to the endosperm. Cells of barley starchy endosperm have been described to be dead at later stages of grain development (Domínguez and Cejudo, 2014). The expression of the *HvMAN3-6* genes, although lower than that of *HvMAN1*, is detected specially in the germinating endosperm. In **Figure 5**, it is shown that MAN activity progressively increases both in the embryo and in the endosperm of germinating grains getting a maximum at 48–72 h, after coleorhiza and root protrusion (t_CE50_ = 24 h; t_RE50_ = 36 h). Since *HvMAN* expression profiles and MAN hydrolytic activity do not temporally match, some post-translational modifications of the HvMAN1 protein cannot be ruled out. This has also been suggested for other MAN proteins, such as LeMAN4 of tomato (Schröder et al., 2006). Mannan polymers are highly abundant in the endosperm CWs at early germination, but they tend to disappear at 42 h, coinciding with the peak of MAN activity in the endosperm and in the embryo. Considering that the HvMAN1 protein sequence has a typical signal peptide (**Supplementary Table S1**, Hrmova et al., 2006) and its mRNA is localized to the AL, it could be possible that HvMAN1 moved *via* the apoplast from the AL to the endosperm where the mannans are to be hydrolyzed. In *A. thaliana*, the movement of the AtMAN7 protein through the apoplast has been previously demonstrated by transient assays in onion epidermal cells (Iglesias-Fernández et al., 2013). Similar transient expression assays have been performed to demonstrate the apoplastic movement of the MAN PtrMAN6 of *Populus trichocarpa* (Zhao et al., 2013). Protein secretion to the apoplast is essential to deal with diverse cell and physiological processes such as CW modification and defense response (Chung and Zheng, 2017). In *Hordeum vulgare*, it has also been proposed that starch mobilization requires a previous CW degradation in order to allow a rapid diffusion of amylolytic enzymes (Andriotis et al., 2016). In the Brassicaceae *Sisymbrium officinale* and *Brassica rapa*, mannans and *MAN* genes have been localized to different seed compartments, again suggesting the movement of MAN enzymes from the embryo tissues to the mucilage layer where mannans are localized and hydrolyzed (Carrillo-Barral et al., 2018). Taking into account the MAN enzymatic activity in embryos and endosperms of germinating grains (**Figure 5B**), the highest expression levels of *HvMAN1* and the mannan polymers disappearance upon germination, a possible role for HvMAN1 can be anticipated, without excluding the activity of other MANs (HvMAN3, HvMAN5, and HvMAN6).

Mannans, glucomannans, and galactomannans have been previously described as CW storage polysaccharides in several species from diverse families, such as Poaceae, Leguminosae, and Solanaceae (Meier and Reid, 1982; Buckeridge, 2010; Guillon et al., 2012). In fenugreek seeds, galactomannans present at the endosperm CWs play a role as storage components, with a yield of ~30% of the seed dry weight (Salarbashi et al., 2019). *LeMAN1* has been involved in the mobilization of mannans localized to the lateral endosperm of *Solanum lycopersicum* seeds, being the hydrolytic product used to nourish the growing embryo (Nonogaki et al., 2000; Lee et al., 2012). MAN enzymatic activity has been also associated with the mobilization of reserve compounds during *Oryza sativa* grain germination (Ren et al., 2008). Mannan polysaccharides contained in the endosperm CWs of *Brachypodium distachyon* grains have been described not only as structural polysaccharides, but also as storage compounds, and the MAN encoding genes *BdMAN4* and *BdMAN6* are specifically expressed at the aleurone cells (Guillon et al., 2012; González-Calle et al., 2015).

Taken together, these data indicate that mannans, deposited in the barley starchy endosperm CWs during grain development, besides their structural function, could be used as reserve compounds upon barley post-germination where MAN activity is highly intense. In this respect, the mRNA expression analysis and the patterns obtained by *in situ* hybridization assays suggest that HvMAN1 synthesized in the AL during early grain imbibition could later move *via* the *apoplast* to hydrolyze the endosperm CW mannan polymers after germination *sensu stricto*.

## Data Availability Statement

All datasets for this study are included in the article/**Supplementary Material**.

## Author Contributions

RI-F has conceived the main idea of the study. RI-F and EP-M have performed the experiments. RI-F, PC, and JV-C have designed the experiments and written the paper.

## Funding

We greatly thank Prof. Ángel Matilla for critical reading of the manuscript. This work was financially supported by grants BIO2017-82873-R (p.i.: JV-C and RI-F) from MINECO-Spain and VJIDOCUPM19ECB form UPM (p.i.: RI-F). EP-M was the recipient of a *Technical Fellowship* from *Comunidad Autónoma de Madrid (*PEJ16/BIO/TL-1451) - APOYO-JvENEs-MT0KUE-25-MKITFK (p.i. R.I-F).

## Conflict of Interest

The authors declare that the research was conducted in the absence of any commercial or financial relationships that could be construed as a potential conflict of interest.
