# Supplementary material for: A Possible Role of the Aleurone Expressed Gene HvMAN1 in the Hydrolysis of the Cell Wall Mannans of the Starchy Endosperm in Germinating Hordeum vulgare L. Seeds
Source: Front Plant Sci. 2020 Jan 20;10:1706. doi: 10.3389/fpls.2019.01706 (PMC6983769; doi:10.3389/fpls.2019.01706)
Supplement: Supplementary file 1 [file DataSheet_1.pdf]

Supplementary Figure S1

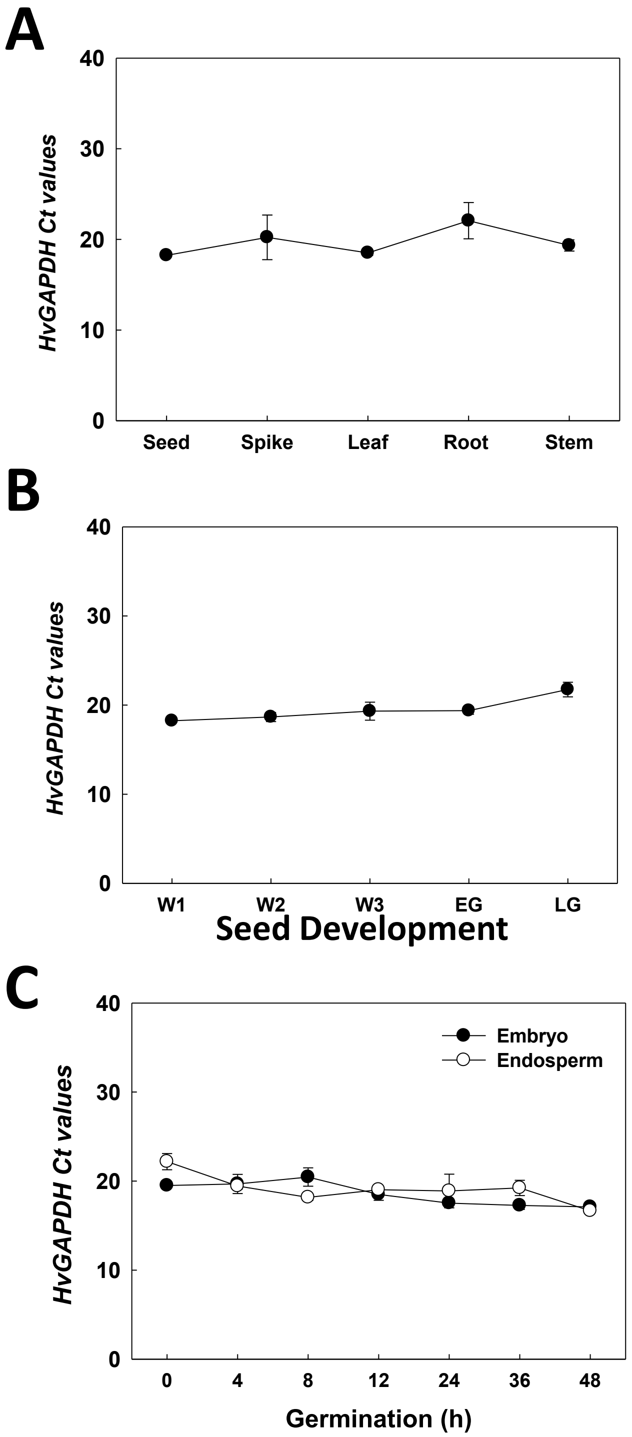

Supplementary FIGURE S1: Transcription levels (Ct mean values) of the *HvGAPDH* gene, housekeeping gene used to normalize the quantitative PCR data, **(A)** in different vegetative (leaf, root, stem) and reproductive (seed, spikes) organs, **(B)** upon seed development and **(C)** at different time point of germination (0, 4, 8, 12, 24, 36, 48 h). White 1: W1; White 2: W2; White 3: W3; Early Green: EG; Late Green: LG.

Supplementary Figure S2

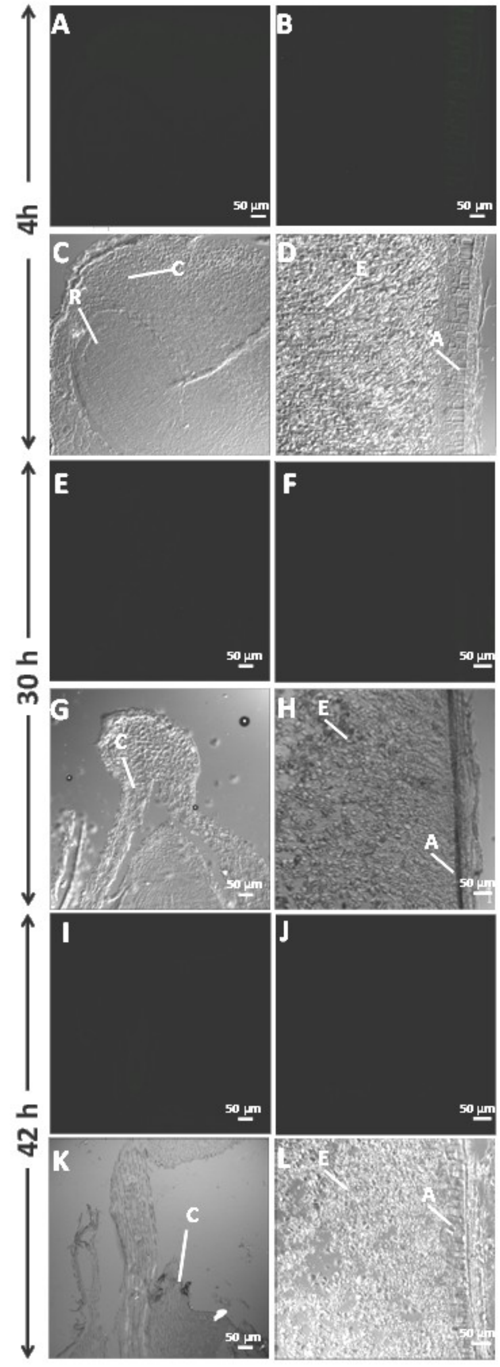

Supplementary FIGURE S2: Secondary Antibody negative controls of sections presented in Figure 6. **(A-D)** 4 h of seed germination. **(E-H)** 30 h of seed germination. **(I-L)** 42 h of seed germination. **(C-D,G-H, K-L)** DIC images. AL: Aleurone Layer; C: Coleorhize; E: Endosperm; R: Root.
