# Supplementary material for: A Possible Role of the Aleurone Expressed Gene HvMAN1 in the Hydrolysis of the Cell Wall Mannans of the Starchy Endosperm in Germinating Hordeum vulgare L. Seeds
Source: Front Plant Sci. 2020 Jan 20;10:1706. doi: 10.3389/fpls.2019.01706 (PMC6983769; doi:10.3389/fpls.2019.01706)
Supplement: Supplementary file 2 [file DataSheet_2.pdf]

A possible role of the aleurone expressed gene *HvMAN1* in the hydrolysis of the cell wall mannans of the starchy endosperm in germinating *Hordeum vulgare* L. seeds

Raquel Iglesias-Fernández[1*], Elena Pastor-Mora[1], Jesús Vicente-Carbajosa[1], Pilar Carbonero[1],

[1]Centro de Biotecnología y Genómica de Plantas-Severo Ochoa (CBGP, UPM-INIA). Universidad Politécnica de Madrid (UPM) - Instituto Nacional de Investigación y Tecnología Agraria y Alimentaria (INIA). Campus de Montegancedo, Pozuelo de Alarcón, Madrid 28223.

e-mail corresponding author: raquel.iglesias@upm.es

Supplementary Table S1. Major biochemical characteristics of the deduced protein MAN sequences of *Hordeum vulgare*, *Arabidopsis thaliana* and *Brachypodium distachyon*.

Protein name	Locus/Ref.	Protein size (aa)	Ip	Mw (Da)	SP (position)
**HvMAN1**	*HORVU3Hr1g060060/ AK356724*	427	9.20	46077.81	YES (25-26 aa)
**HvMAN2**	*HORVU3Hr1g071210/ AK356773*	444	5.85	49902.57	NO
**HvMAN3**	*HORVU5Hr1g115310/ AK371032*	480	6.07	53214.95	YES (19-20 aa)
**HvMAN4**	*HORVU0Hr1g017220/ AK373804*	433	6.72	47524.71	YES (30-31 aa)
**HvMAN5**	*HORVU5Hr1g046370/ AK370093*	420	4.61	46964.79	NO
**HvMAN6**	*HORVU7Hr1g045910/ AK369260*	437	8.09	49398.32	YES (22-23 aa)
**AtMAN1**	*At1g02310*	411	5.68	46290.39	Iglesias-Fernández *et al.*, 2011
**AtMAN2**	*At2g20680*	433	6.47	49558.31	
**AtMAN3**	*At3g10890*	414	8.41	46461.86	
**AtMAN4**	*At3g10900*	408	6.67	45376.48	
**AtMAN5**	*At4g28320*	431	8.38	49898.87	
**AtMAN6**	*At5g01930*	448	5.95	50622.24	
**AtMAN7**	*At5g66460*	431	9.33	48572.98	
**BdMAN1**	*Bd2g45790*	417	8.60	45098.78	González-Calle *et al.*, 2015
**BdMAN2**	*Bd2g49682*	446	6.24	49945.77	
**BdMAN3**	*Bd1g32107*	468	5.67	51765.15	
**BdMAN4**	*Bd3g57290*	413	6.84	45112.05	
**BdMAN5**	*Bd4g44340*	389	4.44	43266.27	
**BdMAN6**	*Bd1g42770*	436	8.75	49158.23	

Supplementary Table S2: Characteristics and sequences of the primers used for quantitative PCR experiments

Gene	Primer Name	Sequences (5´-3´)	Amplicon size (bp)	Eff.(%)	Diss. T (ºC)
HvMAN1	*S-HvMAN1-RT (RIF-53)*	AGACCACCACGACAACAAAG	77	80.4	72.4
	AS-HvMAN1-RT (RIF-54)	AAGGCACGCATTCTTACTCC			
HvMAN2	*S-HvMAN2-RT (RIF-55)*	ACATGGAGGAGTTCCACGAC	128	70.5	84.7
	AS-HvMAN2-RT (RIF-56)	GATCCTCTTGGCCTCTTGTTC			
HvMAN3	*S-HvMAN3-R (PCZ109)*	TGCTCATCCTTACGTCCAAAG	87	86	74.1
	AS-HvMAN3-RT (PCZ110)	ACACGGGACCAATCAAACAG			
HvMAN4	*S-HvMAN4-RT (PCZ111)*	GGCTACGAAGTCGTGCTTG	92	95.13	84.1
	AS-HvMAN4-RT (PCZ112)	TAGCCTCAGGGCGTGATTC			
HvMAN5	*S-HvMAN5-RT (PCZ113)*	CATGGACGATGGTTATGCAG	99	100	74.8
	AS-HvMAN5-RT (PCZ114)	GCAGCATGCTCTCAACATTC			
HvMAN6	*S-HvMAN6-RT (PCZ115)*	AGAGACCCTGACTGGCAATG	72	100	74.8
	AS-HvMAN6-RT (PCZ116)	AACCAGGATTTCCCGGTATC			
HvGAPDH	*S-HvGAPDH-RT (PCZ117)*	GGGAAAGCTCAAGGGTATC	161	100	78.7
	AS-HvGAPDH-RT (PCZ118)	TGTAACCCCACTCGTTGTCA			

Supplementary Table S3. List of primers used for *HvMAN1* probe synthesis in the mRNA *in situ* hybridization analyses

Gene	PRIMERS	SEQUENCES (5´-3´)	Probe size (bp)
HvMAN1	*S-HvMAN1-IS (RIF57)*	ACGCCAAGATCTACCAGTCG	170
	S-HvMAN1-IS (RIF58)	CTTGAGCTTGCGGCTCTC	
